# Short Double-Stranded DNA (≤40-bp) Affects Repair Pathway Choice

**DOI:** 10.3390/ijms241411836

**Published:** 2023-07-23

**Authors:** Zhentian Li, Ya Wang

**Affiliations:** Department of Radiation Oncology, Winship Cancer Institute, Emory University School of Medicine, Atlanta, GA 30322, USA; zhentian.li@gmail.com

**Keywords:** length of small dsDNA, DSB repair, cNHEJ, HR, aEJ, high-LET radiation

## Abstract

To repair ionizing radiation (IR)-induced double strand breaks (DSBs), mammalian cells primarily use canonical non-homologous end-joining (cNHEJ), the homologous recombination (HR) pathway, and the alternative non-homologous end-joining (aEJ) as a backup. These pathways function either compensatively or competitively. High linear energy transfer (LET) compared to low-LET IR kills more cells at the same doses by inhibiting only cNHEJ, but not HR or aEJ. The mechanism remains unclear. The activation of each repair pathway requires the binding of different proteins to DNA fragments of varying lengths. We previously observed an increased generation of small DNA fragments (≤40 bp) in cells following high-LET IR compared to low-LET IR, suggesting that short DNA fragments were one of the major factors interfering with cNHEJ. To provide direct evidence, here we compare the efficiencies of cNHEJ, HR, or aEJ in repairing DSBs containing 30- or 60-bp fragments in vitro and in cells. We show that only cNHEJ but not HR or a-EJ was inefficient for repairing DSBs with 30-bp fragments compared to 60-bp ones, which strongly supports our hypothesis. These results not only enhance our understanding of the DSB repair pathway choice but also hold potential benefits for protection against high-LET IR-induced damage or improving high-LET radiotherapy.

## 1. Introduction

DNA double-strand breaks (DSBs) are a severe threat to cell survival and genomic integrity. In mammalian cells, DNA DSBs are repaired by two major pathways: canonical non-homologous end joining (cNHEJ) and homologous recombination (HR) [1] (one type of homology-dependent repair), as well as by a backup pathway: alternative non-homologous end joining (aEJ) [2,3]. These pathways are all required to maintain genomic integrity [4,5,6,7]; however, they are also competitive and/or inhibitive for each other [8,9,10], depending on the cell status. The choice of DSB repair pathway in mammalian cells depends on various factors such as the type of DSB inducer, cell cycle status, DNA end structure/end modification, etc. [1,11,12,13,14]. However, it remains unclear whether the length of DNA substrates at the ends of DSBs affects repair pathway choice. Among these DSB repair pathways, cNHEJ is the fastest and simplest, as it relies less on end resection/modification and functions throughout the cell cycle; HR functions are limited to S/G2 phases; both HR and aEJ require longer time to process complicated end resection/modification, etc. [1,3,14]. Although these DSB repair pathways function through distinct mechanisms, they are all initiated by proteins that recognize and bind to DSB ends, which activate individual pathways and recruit associated partners to process and seal the breaks by required enzymes. The length of DNA fragments at the ends of DSBs affects the binding efficiencies of specific proteins to activate individual DSB repair pathways. The Ku70/Ku80 heterodimer, as the initial DNA binding protein for cNHEJ, occupies an approximately 20-bp DNA region [15] at each end of the DSB. This binding activates DNA-PKcs that recruit other partners to complete the joining of breaks [15,16,17]. Although Ku heterodimer could bind to as short as 16 bp DNA fragments, such binding could not induce proper conformation change and could not activate DNA-PKcs as effectively as binding to longer (e.g., 60-bp) DNA fragments [18]. In HR, MRE11, in complex with RAD50/NBS1, binds to approximately an 8-bp DNA region [19] at each end of the DSB, which activates ATM and thus recruits other partners to process/complete HR [14,19]. For aEJ, PARP1 binds to approximately a 6-bp DNA region [20] at each end of the DSB, recruiting other partners for break joining [3].

Ionizing radiation (IR)-induced DSBs (~80%) in mammalian cells are repaired by cNHEJ within the first hour after irradiation [21]. High linear energy transfer (LET) IR (component of space radiation or generated from heavy ion radiotherapy facilities) is more effective at killing cells than low-LET IR (γ- or x-ray) at the same doses, demonstrating a high relative biological effectiveness (RBE). The higher RBE value on cell killing is attributed to high-LET IR inducing more clustered DNA damage, which contains both DNA breaks and base damage in a local area, making it more challenging to repair compared to single damaged DNA [22,23,24]. Interestingly, although wild-type cells and cells deficient in HR or aEJ were more sensitive to high-LET than to low-LET IR [25,26], cNHEJ-deficient cells showed similar sensitivity to high-LET and low-LET IR [25,27,28]. These results indicate that cNHEJ is crucial for high-LET IR-induced high RBE on cell killing. In other words, high-LET IR-induced clustered DNA damage interferes specifically with the fast/simple cNHEJ but not the slow/complicated HR or aEJ. Thus, the main cause for clustered damage to result in inefficient cNHEJ should be simple and occur at an early repair stage, although the mechanism remains to be elucidated.

We previously found that high-LET IR generated more small DNA fragments (≤40 bp) compared to low-LET IR in cells [25]. Another group using atomic force microscopy also discovered the increased short DSB fragments in high-LET irradiated cells [29]. Combining these findings with the knowledge that different initial proteins for activating different DSB repair pathways bind to different lengths of dsDNA fragments, shorter DSB fragments (≤40 bp) in high-LET irradiated cells might be one of the main reasons for cNHEJ being the only inefficient DSB repair pathway in high-LET irradiated cells and thus increasing cell killing [30]. Since MRE11 or PARP1 requires a shorter dsDNA sequence for binding compared to Ku, the small DSB fragments have less impact on the binding efficiency of MRE11 or PARP1. Actually, these short DSB fragments do not affect Ku binding but rather interfere with the proper binding of Ku to both ends of a single fragment simultaneously. To date, the hypothesis has been supported by the data derived from protein structure, increased small DSB fragments, and cell survival. However, direct evidence regarding the impact of small DNA fragment length on individual DSB repair pathways is still lacking. This study aims to directly compare the joining efficiencies of 30- and 60-bp DNA fragments through cNHEJ, HR, or aEJ in vitro and/or in cells. Thus, the results will not only help to understand whether the length of small DSB fragments affects repair pathway choice but also have the practicable potential to guide prevention of high-LET IR-induced damage and improvement of high-LET radiotherapy.

## 2. Results

**Figure 1:**
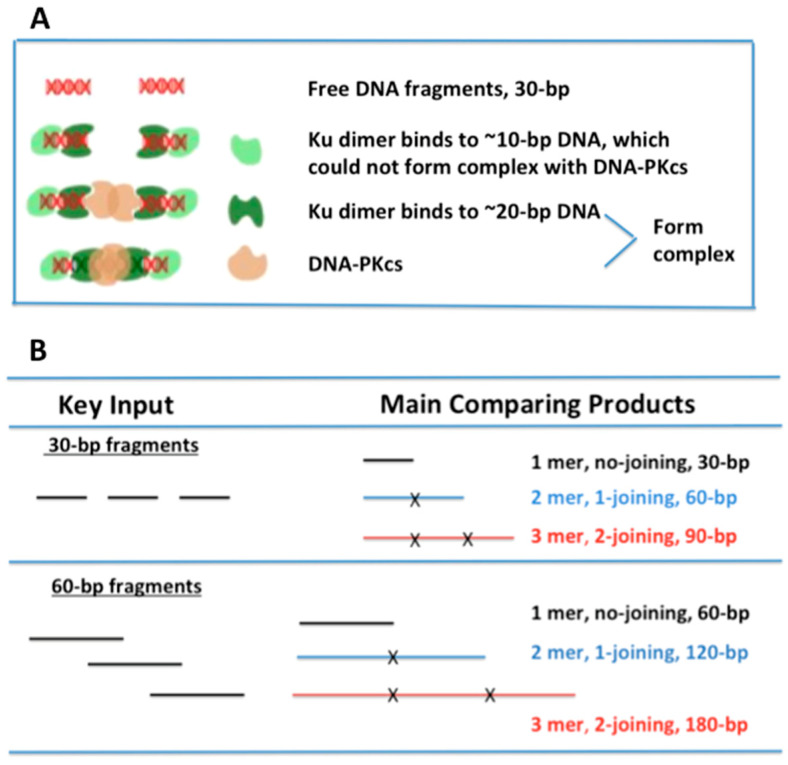
Hypothesis and major comparison of products. (**A**) Hypothesis: 1-joining (2-mer) products for 30-bp DNA fragments were one of the major sources of interference with cNHEJ. One end of the DNA fragment was properly bound by the Ku70/80 dimer (occupied ~20 bp at each end of the DSB), which formed a functional complex with DNA-PKcs. The two functional DNA/Ku/DNA-PKcs complexes from each end of the fragment were then joined together. However, the other end of the fragment had only ~10 bp remaining, lacking sufficient space for proper binding by Ku. As a result, Ku was unable to recruit or activate DNA-PKcs, and this end became a “dead end” that blocked further repair. (**B**) Description of the key input (30- and 60-bp dsDNA) and the major comparison of products for the experiments.

To test whether the length of small dsDNA fragments affects DSB repair pathways differently, we chose 30- and 60-bp dsDNA as the comparison targets. Such a design was based on the following fact: In cNHEJ, Ku binding to DNA ends was in a sequence-independent manner, which shaped an asymmetric ring, encircling the DNA, but also left parts of the helix exposed. The exposed helix is then bound by DNA-PKcs, which is required for cNHEJ [31]. The efficient Ku binding occupies an approximately 20-bp DNA region [15] at each end of DSB, although Ku could bind to dsDNA fragments less than 20 bp at each end of DSB. For example, with 16-bp dsDNA, such binding could not efficiently activate DNA-PKcs [18]. In HR, MRE11 binds to ~8-bp dsDNA [19] at each end of the DSB. In aEJ, PARP1 binds to ~6-bp dsDNA [20] at each end of the DSB. Therefore, we hypothesized that 30-bp DNA fragments did not have enough space for efficient Ku binding to both ends of a DSB fragment (Figure 1A), whereas these short DNA fragments still offered sufficient space for MRE11 or PARP1 binding; thus, the short fragments (30 bp) interfered with only cNHEJ but not HR or aEJ efficiency.

### 2.1. cNHEJ Was Less Efficient in the Joining of 30 bp dsDNA Compared to 60 bp dsDNA In Vitro

**Figure 2:**
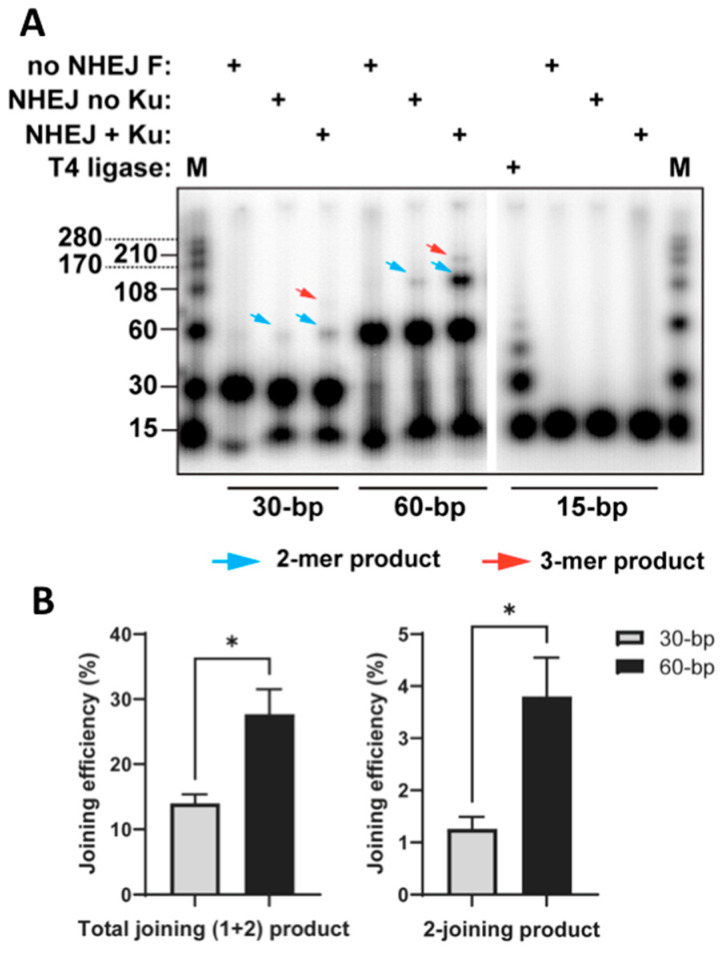
Size dependence of end-joining efficiency through cNHEJ. (**A**) The end-joining reaction was performed as described in Section 4. T4 ligase was used as a positive control. Blue arrow: 2-mer (1-joining) products; red arrow: 3-mer (2-joining) products (see Figure 1B). (**B**) Quantification of end-joining efficiencies. The standard error of the mean was derived from three independent experiments. The efficiency of end-joining was calculated as the percentage of the joined signals to the total signals. Total joining efficiency was calculated as follows: (2-mer + 3-mer)/(1-mer + 2-mer + 3-mer); 2-joining efficiency was calculated as follows: 3-mer/(1-mer + 2-mer + 3-mer). * *p*< 0.05.

Based on the hypothesis (Figure 1A), we designed an in vitro assay to compare the joining efficiency of 30- and 60-bp dsDNA fragments (Figure 1B). We used purified proteins (Ku70/80, DNA-PKcs, Lig4/XRCC4, and XLF) in this experiment. For the experiment, 15-bp DNA fragments were used as a negative control since they could only be joined by T4 ligase and should not be joined by cNHEJ factors. The results show weak signals for the 1-joining product for 30- or 60-bp fragments without Ku as well as the 2-joining product for 30-bp fragments with Ku (Figure 2A). Such unexpected signals were either artificial products or small parts of DNA fragments joined by NHEJ through an unknown Ku-independent mechanism. Importantly, 60-bp fragments exhibited significantly higher efficiencies in both total joining (including 1-joining (2-mer) and 2-joining (3-mer) products) and 2-joining (3-mer) products compared to 30-bp fragments (Figure 2). The 2-joing (3-mer) products represent complete joining, as described in Figure 1B. However, the 1-joining (2-mer) products of 30-bp fragments represent products with “dead ends” to block further cNHEJ, as illustrated in Figure 1A, which is further explained in the Discussion section. These results provide direct evidence that short DNA fragments (~30 bp) are associated with reduced cNHEJ efficiency compared to longer fragments (60 bp).

### 2.2. Construction of the Reporter System with Two Digestion Sites Separated by 30- or 60-bp for HR, cNHEJ, or aEJ Cell Assays

**Figure 3:**
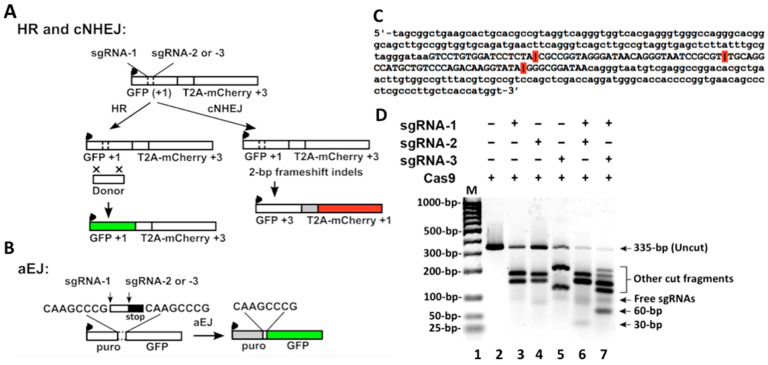
Reporter cassettes containing sgRNAs/Cas9 digestion sites. (**A**) Reporter of the cNHEJ/HR pathway. A promoter was linked to a GFP cDNA at transcriptional reading frame +1 that was interrupted by an intervening sequence containing target sites of sgRNA-1, 2, and 3. A T2A-linked mCherry cDNA was located downstream with a transcriptional reading frame + 3. The arrow denotes the promoter. Co-delivering a donor template with Cas9/sgRNA allowed HR to repair the digested DSBs, which restored GFP expression through gene conversion. (**B**) Reporter of the aEJ pathway. An intervening fragment including three sgRNA target sites followed by a stop codon separated a promoter and a downstream GFP cDNA. The cassette was flanked by two 8-nucleotide homologous sequences. These Cas9/sgRNA-induced DSBs were repaired through the microhomology-mediated aEJ pathway, which activated GFP expression. (**C**) The sequence of DNA substrate used for in vitro Cas9/sgRNA digestion. The sequence in capitals represents one inserted into the two (cNHEJ/HR and aEJ) vectors. The red bars indicate sgRNAs that guided Cas9 digesting sites; the first was with sgRNA-1, the second was with sgRNA-2, and the third was with sgRNA-3. Combining Cas9 with sgRNA-1/2 generated 30-bp dsDNA, and sgRNA-1/3 generated 60-bp dsDNA. (**D**) Cas9/sgRNA digestion products. These sgRNAs were designed using the CHOPCHOP toolbox. Detailed information is provided in Section 4.

HR and aEJ are relatively complicated repair processes; they are difficult to reconstitute biochemically (particular HR) using an in vitro system compared to cNHEJ. Therefore, we used engineered reporter systems to compare these pathways in cells. Although we previously studied HR efficiency in human and mouse cells using the I-SceI-based reporter from Dr. Jasin’s lab [32,33,34], the vector did not meet our requirements in this study. We chose a lentiviral reporter vector (pCVL Traffic Light Reporter 1.1 (Sce target) Ef1a Puro (Addgene #31482)) generated by Dr. Scharenberg’s lab [35]. This vector was designed to examine two DSB repair pathways simultaneously by detecting green and red fluorescence signals. We modified this vector for the cNHEJ/HR assay, as indicated in Figure 3A. For aEJ, we modified the EJ2GFP-puro vector (Addgene #44025), as indicated in Figure 3B. The original aEJ vector was generated by Dr. Stark’s lab [36]. The modified constructs utilized CRISPR/Cas9 [37] to induce DSBs. We inserted a dsDNA oligo sequence that had minimal homology to the human genome into the I-SceI digest sites of the original reporter vectors (Figure 3A,B). The inserted sequence contained three single guide RNA (sgRNA) recognizing sites for Cas9 digestion: sgRNA-1, sgRNA-2, and sgRNA-3. Combining Cas9 with sgRNA-1 and sgRNA-2 (sgRNA-1/2) generated 30-bp fragments, and with sgRNA-1 and sgRNA-3 (sg-RNA-1/3) generated 60-bp fragments. After modifying the aEJ vector (Figure 3B), we cloned the cassette into a pRRL lentiviral vector backbone (Addgene #12252).

To test the Cas9/sgRNA digestion efficiency for the inserted sequence, we used PCR to amplify the fragment with an adjacent sequence from the cNHEJ/HR vector and generated a 335-bp DNA substrate (Figure 3C). We then performed an in vitro assay using Cas9 with sgRNAs to digest the DNA substrate. Cas9 alone without sgRNA did not show any activity, but combining Cas9 with individual sgRNA (1, 2, or 3) shows that the digestion signals dramatically increased while the uncut signals decreased (Figure 3D). These data indicate that Cas9 digestion completely depends on the sgRNAs. The uncut signals were further reduced when Cas9 combined sgRNA-1/2 or sgRNA-1/3 (Figure 3D), indicating that the sgRNA combination increased Cas9 digestion efficiency. Cas9 with sgRNA-1/2 generated 30-bp signals, and Cas9 with sgRNA-1/3 generated 60-bp signals (Figure 3D). The results indicate that Cas9 with the combined sgRNAs digested the two sites (two cuts) in the DNA substrate. However, in addition to the 30- or 60-bp (two cut) products, there were still residual longer (one cut) products in the Cas9 combined double sgRNAs groups (Figure 3D). These data indicate that when the two-cut products were generated by Cas9/sgRNAs-1/2 or Cas9/sgRNAs-1/3, longer one-cut products were also generated at the same time. Such unexpected results from in vitro digestion should also be inevitable for Cas9/sgRNAs-1/2 or Cas9/sgRNAs-1/3 digestion in cells, as described in the following results.

### 2.3. cNHEJ, Not HR or aEJ, Was Less Efficient for Joining 30-bp than 60-bp dsDNA

We generated new HEK293T cell lines by stably expressing a single copy of the cNHEJ/HR or aEJ lentiviral vector, respectively. We named the cell line that was integrated with the cNHEJ/HR vector HEK293T-1, and the cell line with the aEJ vector HEK293T-2. To compare the repair efficiencies of 30- and 60-bp DSBs in cells using cNHEJ/HR or aEJ assays, we generated 6 groups of transient transfection samples (Table 1) for HEK293T-1 and HEK293T-2 cell lines, respectively. The only difference in sample preparation between HEK293T-1 (cNHEJ/HR) and HEK293T-2 (aEJ) cell lines was the inclusion of the donor vector in HEK293T-1 but not in the HEK293T-2 cell line.

**Figure 4:**
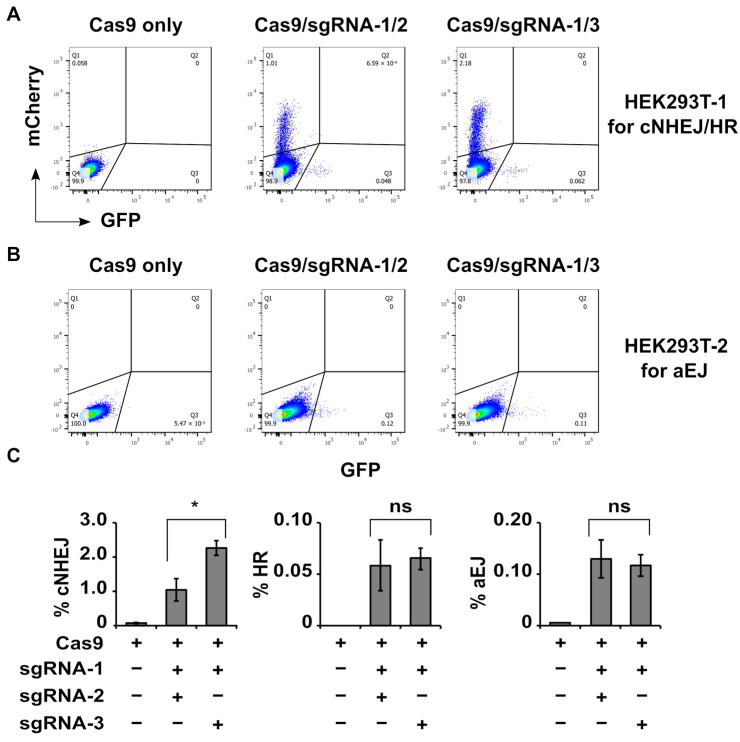
cNHEJ, but not HR or aEJ, show less efficiency for joining 30-bp than 60-bp dsDNA. (**A**) The flow cytometry plots were obtained from HEK293T-1 cells transduced with Cas9/sgRNAs (Table 1) plus donor vector. mCherry signals (in the Q1 area) represent cNHEJ of the Cas9/sgRNA-digested DSBs, and GFP signals (in the Q3 area) represent HR repair of the Cas9/sgRNA-digested DSBs. The signals in the Q2 area represent both mCherry and GFP positive signals that should not exist and were not present in this study. The Q4 signals represent those sorted cells without mCherry or GFP signals. Complete data are shown in Table 2, Figures S1 and S2, and Table S1. (**B**) GFP signals in the Q3 area represent aEJ of the Cas9/sgRNA-digested DSBs. Complete data are shown in Table 2, Figures S3 and S4, and Table S2. (**C**) Quantification of the relative efficiencies of cNHEJ, HR, or aEJ in repairing Cas9 with sgRNA-1/2-induced DSBs (containing 30 bp) and Cas9 with sgRNA-1/3-induced DSBs (containing 60 bp). The data represent the mean ± standard deviation from Table 2, Table S1 and Table S2. * *p* < 0.05; ns, no significant difference.

The results show that group 1 of HEK293T-1 or HEK293T-2 cells did not generate clear DSB repair signals (panel 1 in Figure 4A,B). Groups 2–4 resulted in clear mCherry (cNHEJ) as well as GFP (HR) signals in HEK293T-1 cells and GFP (aEJ) signals in HEK293T-2 cells (Table 2). The analyzed data shown in Table 2 were the mean ± standard deviation from samples in triplicate that were shown in Figures S1–S4 and Tables S1 and S2. The mCherry (cNHEJ) signals were stronger than GFP (HR) signals in HEK293T-1 cells (Table 2), indicating that cNHEJ was much more efficient than HR in repairing Cas9-induced blunt-end DSBs in the cells predominantly in G1-phase. The GFP signals in HEK293T-2 cells mainly reflected aEJ efficiency (Table 2) since this modified reporter was derived from the original EJ2GFP-puro vector with less influence from cNHEJ [36] These data indicate that, similar to the in vitro digestion data (Figure 3D), Cas9-induced DSBs were completely dependent on sgRNA, and the cells are capable of repairing the one-cut DSBs.

Also, similar to the in vitro data (Figure 3D), groups 5 and 6 of HEK293T-1 or HEK293T-2 cells should generate 30- or 60-bp (two-cut) DSB fragments as well as one-cut ones. Such one-cut products had no length problem for proper binding by Ku; they could be efficiently repaired by cNHEJ. Therefore, a relatively large portion of mCherry (cNHEJ) signals in Cas9 with sgRNA-1/2 or sgRNA-1/3 (containing 30- or 60-bp DSBs) transduced HEK293T-1 cells (Figure 4A, Table 2) likely represent cNHEJ of the one-cut products. In the current study, we could not separate the two-cut and the one-cut products from the cell data. This indicates that the shown efficiency for cNHEJ of longer one-cut products might cover the inefficiency for cNHEJ of 30-bp (two-cut) products. However, even considering this, the efficiency for cNHEJ of Cas9 with sgRNA-1/2-generated total products (containing 30 bp and longer DSBs) was still significantly lower than that for cNHEJ of Cas9 with sgRNA-1/3-generated total products (containing 60 bp and longer DSBs) (Figure 4C, Table 2). In the same situation, there was no significant difference in the efficiency of HR or aEJ to repair these DSBs (Figure 4C, Table 2). By combining these data with the data shown in Figure 2, we conclude that the 30-bp small DSB fragments mainly interfere with the efficiency of cNHEJ but not that of HR or aEJ.

## 3. Discussion

In this study, we show for the first time that small dsDNA (30 bp) compared to the longer one (60 bp) interfered with cNHEJ but did not affect HR or aEJ efficiency. These results provide direct evidence to support the idea that increased small DSB fragments in high-LET irradiated cells are one of the major reasons for increased cell killing, which well explains previous findings [25,26,27,28,38,39] that high-LET IR primarily inhibits cNHEJ but does not affect HR or aEJ efficiency. Additionally, considering the observed abundance of mCherry signals (indicative of cNHEJ) in the Cas9 with sgRNA-1/2 digested 30-bp experiment (Figure 4 and Table 2), we speculate that non-simultaneous digestion of Cas9 may be another contributing factor.

Different from IR, which simultaneously generates DSBs in exposed cells, DSBs induced by external Cas9 in transduced cells through digesting the integrated two adjacent target sites (close to each other) are impossible to generate simultaneously. In vitro Cas9 digestion should be more likely to be simultaneous than cell Cas9 digestion. As shown in Figure 3D, Cas9 with combined sgRNA-1/2 or 1/3 digested samples contained both one-cut and two-cut products in the gel, well demonstrating the non-simultaneous digestion. Among the non-simultaneous digestions, one may occur delayed relative to the other by several minutes to a longer time. In cells, the half time for the fast components of cNHEJ ranged from 7 to 14 min [40]. Thus, cNHEJ in cells could quickly join not only one-cut DSBs but also the early digested sites from the two-cut DSBs. If the delayed time was long enough, the later digested site could be one DSB without the length’s influence on proper Ku binding. In addition, as shown in Figure 2, there might be additional Ku-independent NHEJ (non-aEJ) to repair a small portion of DSBs in cells, which needs future investigation.

It is worth mentioning that the mCherry signals (cNHEJ) in Cas9/sgRNA-2 transduced HEK293T-1 cells were much higher than those in Cas9/sgRNA-1 or Cas9/sgRNA-3 transduced cells (Table 2, Figure S2), indicating more efficient Cas9/sgRNA2 digestion in cells in vitro. Meanwhile, both the GFP signals (HR for HEK293T-1 and aEJ for HEK293T-2 cells) with Cas9/sgRNA-2 transduction were relatively lower than those with Cas9/sgRNA-1 or Cas9/sgRNA-3 transduction (Table 2, Figures S2 and S4). These observations suggest that there may be competition from more efficient cNHEJ in these cells, although HEK293T-2 cells did not show cNHEJ signals. The mechanism underlying the different digestion efficiencies between Cas9/sgRNA-2 and Cas9/sgRNA-1 or -3 remains unclear. However, the highly effective digestion of Cas9/sgRNA-2 in cells indicates that Cas9/sgRNA-2 plus sgRNA-1 should induce more 30-bp DNA fragments. Even though there should also be more one-cut products, the cells still show a significantly lower cNHEJ ratio for repairing 30-bp versus 60-bp DSBs (Figure 4C). These results excluded the possibility that the different cNHEJ efficiencies were due to the different digestion ratios. Thus, these results strongly support that the significantly lower cNHEJ efficiency for repairing 30-bp versus 60-bp DSBs (Figure 4C) was due to the short length of the fragments.

In human cells, Ku molecules are in high abundance, and with an extremely high affinity for DNA ends, Ku can immediately bind to DSB properly (≥20 bp) and improperly (≤16 bp). However, since Ku dissociating from bound DNA required DNA-PKcs activation [41], the improperly bound Ku could not recruit DNA-PKcs [18], leading to their retention at the ends and rendering them non-functional “dead ends” that impeded further repair, as illustrated in Figure 1A. High-LET IR increased the generation of short DSB fragments (≤40 bp) [25], which increased the Ku-improperly bound “dead ends”, hindering efficient cNHEJ. The results shown in this study, supported by the findings from other groups [18,41], suggest that the short DSB fragments (≤40 bp) that formed Ku-dependent “dead ends” not only interfered with cNHEJ, but could also be used to affect the efficiency of HR or aEJ. For example, DNA-PKcs inhibitors that were initially designed to inhibit cNHEJ only now, based on our results, could also inhibit phosphorylation and dissociation of Ku from bound DNA, which in turn blocked MRE11 or PARP1 from competitively binding to the damaged DNA ends.

Without cNHEJ, high-LET IR-induced increased short DNA fragments can still be repaired by HR and/or aEJ. Such a statement is strongly supported by the results derived from synchronized S-phase Ku-deficient cells that showed the same sensitivities to high-LET and low-LET IR-induced killing [42], despite the increased short dsDNA fragments induced by high-LET IR [25]. These results were consistent with data from the DT40 cells [28], a chicken lymphoma cell line with highly efficient HR due to the high ratio of S-phase cells from a short cell cycle. Ku-knockout DT40 cells showed significant resistance to both low-LET and high-LET IR at the same levels compared to their wild-type counterparts [28]. These results highlight that the increased small DSB fragments induced by high-LET IR did not affect HR or aEJ efficiency, supporting the notion that the length of these fragments has a limited impact on the efficient binding of MRE11 or PARP1. Therefore, high-LET IR-induced short DNA fragments that create Ku-dependent “dead ends”, might be one of the key events resulting in the high RBE on cell killing. In the future, it is still necessary to develop new assays to verify this hypothesis by directly showing that cNHEJ cannot efficiently repair ≤ 40-bp dsDNA fragments (not in a mixture) in cells.

Although cNHEJ-deficient cells showed equal sensitivities to the same doses of high-LET and low-LET IR, cNHEJ proficient cells still exhibit greater resistance than cNHEJ deficient cells following high-LET IR exposure [25,28], indicating that partial high-LET IR-induced DSBs were repaired by cNHEJ. This is because of the long DSB fragments that are the majority of DSBs induced by IR, including high-LET IR in cells [25]. cNHEJ as the main pathway could efficiently repair the long DSBs, including those > 40-bp fragments. Only the “dead ends” resulting from the improper binding of Ku to small DSB fragments in high-LET irradiated cells (as depicted in Figure 1A) remain unrepaired, thereby representing one of the major reasons for the high RBE observed in cell killing.

## 4. Materials and Methods

### 4.1. In Vitro c-NHEJ Assay

DNA ladder (marker) preparation: 15-, 30-, 60-, and 108-bp dsDNA oligoes with blunt ends were synthesized by Integrated DNA Technologies (IDT) Inc. (Coralville, IA) Longer dsDNAs (170, 210, and 280 bp) were PCR amplified from pRRL sEF1a HA.NLS.Sce(opt).T2A.IFP (#31484, Addgene, Watertown, MA). The dsDNA sequences are as follows: 5′-TAGAG ACGGGATGAG-3′ for 15 bp; 5′-TAGAGACGGGATGAGTGGAATTAGGACTGA-3′ for 30 bp; 5′-TAGAGACGGGATGAGTGGAATTAGGACTGAGA CTATGGTTGCTGACTAATCGAGACCCAT-3′ for 60 bp; and 5′-GCGTAGGGATAACAGGGTAATTAGATGATAGAGACGGGATGAGTGGAATTAGGACTGACGCG GGCAATCACTCGAGTGAGTAACTAGCAAGCCCGGCGTGGATCCGC-3′ for 108 bp. The PCR products were obtained using one shared primer, “CGGAATTCAAGACCTACTGCATGCAGT”, and the other primers “CGGAATTCTGAAGGTCTGGGCGCC” for 170 bp; “CGGAATTCGGTCTTCTTGTTGTTCACGAT” for 210 bp; and “CGGAATTCCCGCCG TCGTCCATGA” for 280-bp. These dsDNA fragments were labeled with ATP, [γ-32P] (PerkinElmer), using T4 Polynucleotide Kinase (T4 PNK) (EK0031, Thermo Scientific, Waltham, MA, USA), and then purified with a G-25 spin column (Microspin™ G-25 Columns, GE Healthcare, 27-5325-01, Chicago, IL, USA).

For the end-joining reaction, DNA-PKcs were purchased from Thermo Fisher Scientific Inc. (PV5866). The other purified proteins, including Ku dimers Lig4/XRCC4 and XLF, were obtained from Dr. William Dynan’s lab [43,44,45]. The end-joining reactions were performed using 50 nM of Ku70/80, 25 nM of DNA-PKcs, 100 nM of Lig4/XRCC4, 100 nM of XLF, and 0.5 nM [γ-^32^P] labeled dsDNA fragments (15, 30, or 60 bp), with 5 mM triethanolamine-HCl, 10 mM Tris-HCl, 50 mM Mg(OAc)2, 0.1 mM DTT, 50 ng/µL BSA, and 10 nM ATP at 37 °C for 30 min. T4 ligase was used as a positive control. The reaction products were resolved in a 12.5% (1.5 mm thick) denaturing polyacrylamide gel. The image of the gel with the radio-activated signals was obtained using a Typhoon laser scanner.

### 4.2. Construction of Reporter Vectors

Standard molecular biology techniques were used for plasmid construction. A dsDNA oligo that had minimal homology to the human genome was synthesized, containing targeting sites for sgRNA-1 (GTCCTGTGGATCCTCTACGC), sgRNA-2 (GGACAGCATGGCCTGCAACG), and sgRNA-3 (GTCCCAGACAAGGTATAGGG), so that co-expression of Cas9 with sgRNA-1 and 2 resulted in a 30-bp fragment, and co-expression of Cas9 with sgRNA-1 and 3 resulted in a 60-bp fragment. The HR/cNHEJ reporter psf.CVL Traffic Light Reporter Ef1a Puro was modified from the lentiviral vector pCVL Traffic Light Reporter 1.1 (Sce target) Ef1a Puro (Addgene #31482) by inserting the above synthesized dsDNA fragment into the original I-SceI recognition site. Briefly, a promoter was linked to a GFP cDNA at transcriptional reading frame +1 that was interrupted by an intervening sequence (the capital ones shown in Figure 3C) containing sgRNA target sites. A T2A-linked mCherry cDNA was located downstream with a transcriptional reading frame + 3. A 2-bp frame shift activated T2A-linked mCherry expression that represents cNHEJ, since cNHEJ products were frequently accompanied by small indels and thus caused transcriptional reading frame shifts. HR repair of Cas9/sgRNA-induced DSBs was processed by co-delivering a donor template (pRRL SFFV d20GFP.T2A.mTagBFP Donor (Addgene #31483)), which restored GFP expression through gene conversion. The aEJ reporter pRRL-sfGFP-aEJ-puro was modified from the original non-lentiviral vector, EJ2GFP-puro (Addgene #44025), by inserting the synthesized dsDNA fragment described above into the original I-SceI site. The reporter was then transferred to the lentiviral backbone of plasmid pRRL-sEF1a-HA.NLS.Sce (opt). T2A.IFP (Addgene #31484). The reporter cassette contains a promoter and a downstream GFP cDNA that were separated by an intervening fragment including 3 sgRNA target sites (the sequence in capital shown in Figure 3C), followed by a stop codon, flanked by two 8-nucleotide homologous sequences. Micro-homology-mediated repair of Cas9/sgRNA-induced DSBs restores GFP expression.

### 4.3. Cas9/sgRNA In Vitro Digestion Assay

These sgRNAs were designed using the CHOPCHOP toolbox [46]. The predicted efficiencies were as follows: sgRNA-1, 72.92%; sgRNA-2, 69.75%; and sgRNA-3, 69.46%. The DNA substrate (335 bp) was amplified by PCR using primers 5′-ACCATGGTGAGCAAGGG-3′ and 5′-TAGCGGCTGAAG CACTG-3′ from psf.CVL Traffic Light Reporter Ef1a Puro. To form sgRNAs/Cas9 RNPs, with 2 μM of recombinant Alt-R S.p. Cas9 (purchased from IDT) and 1 μM of sgRNAs (synthesized by IDT), and then incubated with 1 μM Cas9 RNPs with 50 nM of substrate DNA at 37 °C for 1 h. Ladders (Thermal Fisher Scientific Inc., BP2571100) and digested DNA were resolved on a 2% low melting temperature SeaPlaque Agarose gel (Lonza, Switzerland), followed by SYBR Gold Nucleic Acid Gel Stain (Thermal Fisher Scientific Inc.).

### 4.4. Generating Cell Lines

HEK293T cells were maintained in DMEM supplemented with 100 units/mL penicillin, 100 mg/mL streptomycin, and 10% fetal bovine serum. Reporters expressing lentiviral particles for the cNHEJ/HR or aEJ cassette were generated, and HEK293FT cells were transduced. Briefly, 2 × 10^5^ HEK293 cells were transduced with 1 μL of un-concentrated reporter lentivirus (~5% transduction). At 3 days post-transduction, cells with integrated reporters were selected using 1 μg/mL puromycin for 5 days. Puro-resistant cells were selected at the minimal multiplicity of infection (MOI) (surviving fraction < 0.1), and a monoclonal population of cells was isolated and used for further analysis. The cNHEJ/HR reporter stably-expressing cell line was named HEK293T-1, and the aEJ reporter stably-expressing cell line was named HEK293T-2.

### 4.5. Reporter Assay with Flow Cytometry

To generate 30- or 60-bp small dsDNA fragments, sgRNA-1, sgRNA-2, or sgRNA-3 were cloned into the pX330-U6-Chimeric_BB-Cbh-hSpCas9 vector (Addgene #42230) individually or in pairs, sgRNA-1 plus 2 (sgRNA-1/2) or sgRNA-1 plus 3 (sgRNA-1/3). For the cNHEJ/HR assay, the transduction was paired with a donor vector, pRRL SFFV d20GFP.T2A.mTagBFP Donor (Addgene #31483). Cells were harvested 72 h post-transduction and analyzed on a Flow Cytometer (BD FACS LSR II). >30,000 cells were sorted for each sample. GFP was measured using a 488 nm laser for excitation and a 530/30 filter. mCherry was excited using a 561 nm laser and was acquired with a 610/20 filter. The data was quantified using FlowJo software v10.

### 4.6. Statistical Analysis

Error bars represent the standard error, as calculated by dividing the standard deviation of replicates by the square root of the number of total replicates. For normalized data shown in Figure 4C, the percent measured value for cNHEJ, HR, or aEJ following flow cytometric analysis of the percent measured value of mCherry or GFP for the indicated total sorted cells. More than 30,000 cells were sorted for each sample. Metric averages and standard deviations were calculated, and statistical significance was determined by the two-tailed Student’s *t*-test.

## 5. Conclusions

In conclusion, DSBs pose a significant threat to cell survival and genomic stability. Unrepaired DSBs can lead to cell death, while misrepaired DSBs can result in chromosomal translocations, which are implicated in the development of cancer [47]. The current study provides direct evidence to support the idea that small DSB fragments (≤30 bp) interfere specifically with cNHEJ efficiency while having less impact on HR or aEJ efficiency. These results highlight that different lengths of small dsDNA fragments from IR-induced clustered damage affect DSB repair pathways, cNHEJ and HR/aEJ, differently. These results not only enhance our understanding of DSB repair pathway choice but also have the practicable potential to guide the prevention of high-LET IR-induced damage and the improvement of high-LET radiotherapy.

## Figures and Tables

**Table 1:** Sample information for reporter experiments.

Sample #	Cas9	sgRNA-1	sgRNA-2	sgRNA-3
1	+			
2	+	+		
3	+		+	
4	+			+
5	+	+	+	
6	+	+		+

**Table 2:** Repair of DSBs digested by Cas9 with sgRNA in HEK293T-1 or HEK293T-2 cells.

	sgRNA-1	sgRNA-2	sgRNA-3	sgRNA-1/2	sgRNA-1/3	Cell Line
	One-Cut	One-Cut	One-Cut	Two-Cuts *	Two-Cuts **	
cNHEJ	3.83% ± 0.004	8.73% ± 0.015	2.56% ± 0.001	0.93% ± 0.002	2.26% ± 0.0002	HEK293T-1
HR	0.14% ± 0.0004	0.04% ± 0.0001	0.12% ± 5.8 × 10^−5^	0.06% ± 0.0002	0.06% ± 0.0001	HEK293T-1
aEJ	0.15% ± 0.0003	0.06% ± 9.6 × 10^−5^	0.13% ± 0.0003	0.13% ± 0.0004	0.12% ± 0.00002	HEK293T-2

* Containing 30-bp digested products; ** Containing 60-bp digested products.

## Data Availability

All data proposed for the manuscript are available from the leading contact (Y.W.) upon reasonable request.

## Supplementary Materials

The following supporting information can be downloaded at: https://www.mdpi.com/article/10.3390/ijms241411836/s1.

## Abbreviations

Abbreviations: aEJ, alternative non-homologous end-joining; cNHEJ, canonical non-homologous end-joining; dsDNA, double strand DNA; DSB, double strand break; HR, homologous recombination; IDT, Integrated DNA Technologies; IR, ionizing radiation; LET, linear energy transfer; MOI, multiplicity of infection; RBE, relative biological effectiveness; sgRNA, single guide RNA.
