# Supplementary material for: Short Double-Stranded DNA (≤40-bp) Affects Repair Pathway Choice
Source: Int J Mol Sci. 2023 Jul 23;24(14):11836. doi: 10.3390/ijms241411836 (PMC10380458; doi:10.3390/ijms241411836)
Supplement: Supplementary file 1 [file ijms-24-11836-s001.zip › ijms-2461445-supplementary.pdf]

Supplementary Materials

Including Supplemental Figures S1-S4 and Supplemental Tables S1, S2

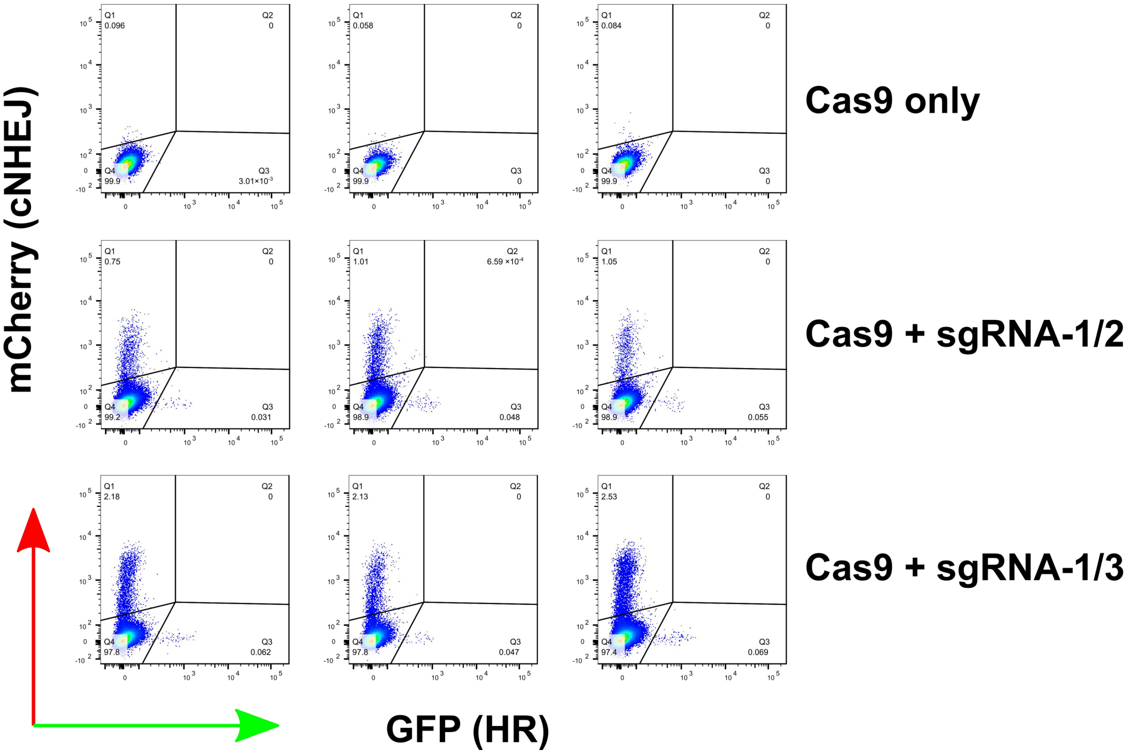

Supplemental Figure S1. Flow cytometry images from Cas9 with or without combined sgRNAs transduced HEK293T-1 cells. Cas9 with combined sgRNAs (1/2 or 1/3) transduced cells generated two-cuts DSBs: sgRNA-1/2 generated 30-bp DSBs; sgRNA-1/3 generated 60-bp DSBs. The mCherry signals in Q1 area represented cNHEJ products. The GFP signals in Q3 area represented HR products. Any signals in Q2 area should represent both mCherry and GFP positive ones that did not exist in this study. The dots accumulated in Q4 area represented those sorted cells without mCherry or GFP signals.

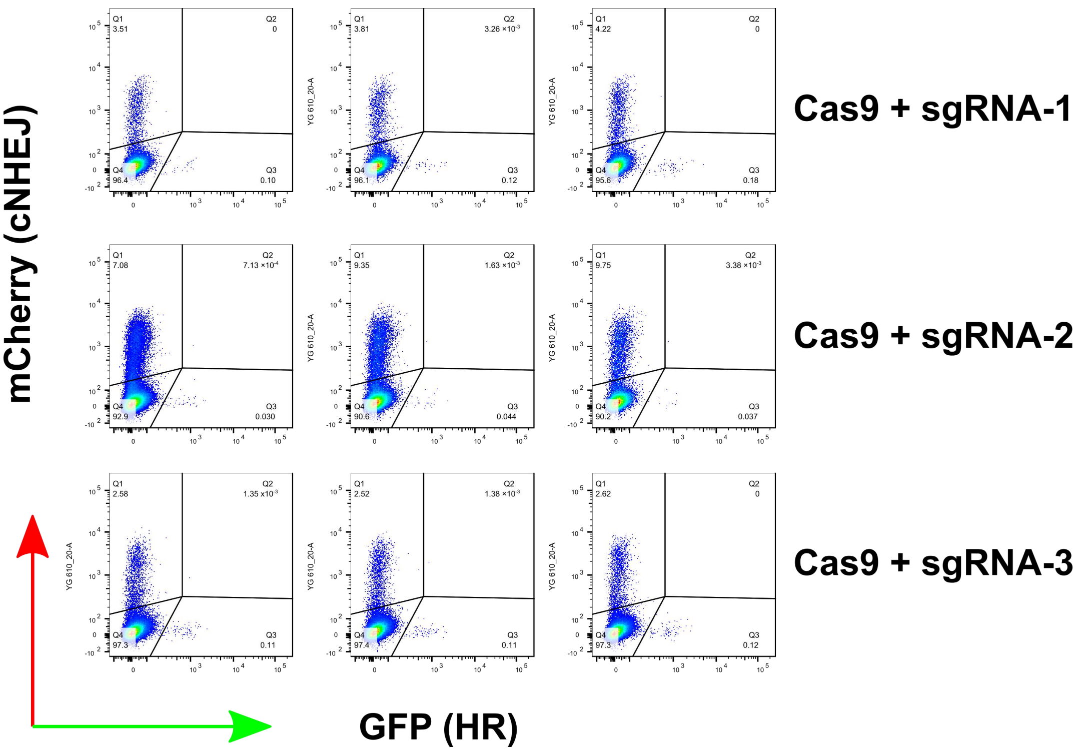

Supplemental Figure S2. Flow cytometry images from Cas9 with individual sgRNA transduced HEK293T-1 cells. Cas9 with individual sgRNA transduced cells generated one-cut DSBs. The mCherry signals in Q1 area represented cNHEJ products. The GFP signals in Q3 area represented HR products. The dots accumulated in Q4 area represent those sorted cells without any mCherry or GFP signals.

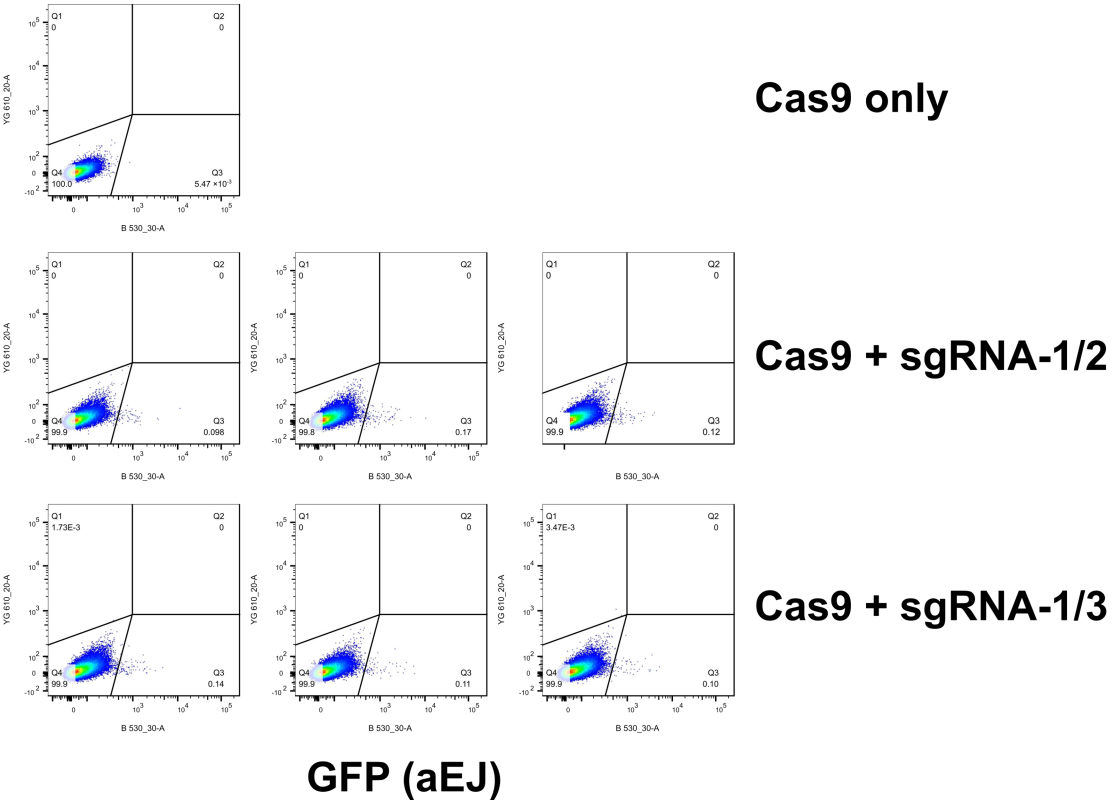

Supplemental Figure S3. Flow cytometry images from Cas9 with or without combined sgRNAs transduced HEK293T-2 cells. Cas9 with combined sgRNAs transduced cells generated two-cuts DSBs: sgRNA-1/2 generated 30-bp DSBs; sgRNA-1/3 generated 60-bp DSBs. The GFP signals in Q3 area represented aEJ products. The dots accumulated in Q4 area represent those sorted cells without GFP signals.

Supplemental Table S1. Analyzing flow cytometry data from HEK293T-1 cells transduced with Cas9/sgRNAs

	P1 | Freq. of Parent	P1/Q1: B 530_30-A- , YG 610_20-A+ | Freq. of Parent	P1/Q2: B 530_30-A+ , YG 610_20-A+ | Freq. of Parent	P1/Q3: B 530_30-A+ , YG 610_20-A- | Freq. of Parent	P1/Q4: B 530_30-A- , YG 610_20-A- | Freq. of Parent
px330_001.fcs	45.5 %	0.10%	0 %	$3.01x10^{-3}$ %	99.9 %
px330_002.fcs	46.3 %	0.06%	0 %	0%	99.9 %
px330_003.fcs	45.8 %	0.08%	0 %	0%	99.9 %
sg1_001.fcs	43.4 %	3.51%	0 %	0.10%	96.4 %
sg1_002.fcs	45.1 %	3.81%	$3.26x10^{-3}$ %	0.12%	96.1 %
sg1_003.fcs	46.9 %	4.22%	0 %	0.18%	95.6 %
sg2_001.fcs	37.1 %	9.75%	$3.38x10^{-3}$ %	0.04%	90.2 %
sg2_002.fcs	43.7 %	7.08%	$7.13x10^{-4}$ %	0.03%	92.9 %
sg2_003.fcs	38.5 %	9.35%	$1.63x10^{-3}$ %	0.04%	90.6 %
sg3_001.fcs	39.0 %	2.58%	$1.35x10^{-3}$ %	0.11%	97.3 %
sg3_002.fcs	42.0 %	2.52%	$1.38x10^{-3}$ %	0.11%	97.4 %
sg3_003.fcs	39.6 %	2.62%	0 %	0.12%	97.3 %
sg12_001.fcs	35.0 %	1.05%	0 %	0.06%	98.9 %
sg12_002.fcs	39.0 %	0.75%	0 %	0.03%	99.2 %
sg12_003.fcs	35.9 %	1.01%	$6.59x10^{-4}$ %	0.05%	98.9 %
sg13_001.fcs	39.4 %	2.18%	0 %	0.06%	97.8 %
sg13_002.fcs	29.1 %	2.13%	0 %	0.05%	97.8 %
sg13_003.fcs	39.4 %	2.53%	0 %	0.07%	97.4 %
	cNHEJ	SD	HR	SD	
vector	0.1%	0.0002	0.00%	0.000000	
SgRNA-1+2 (30 bp)	0.9%	0.0016	0.05%	0.000127	
SgRNA-1+3 (60 bp)	2.3%	0.0022	0.06%	0.000112	
sgRNA-1	3.8%	0.0036	0.13%	0.000416	
sgRNA-2	8.7%	0.0144	0.04%	0.000056	
sgRNA-3	2.6%	0.0005	0.11%	0.000058	

Supplemental Table S2. Analyzing flow cytometry data from HEK293T-2 cells transduced with Cas9/sgRNAs

	P1 | Freq. of Parent	P1/Q1: B 530_30-A- , YG 610_20-A+ | Freq. of Parent	P1/Q2: B 530_30-A+ , YG 610_20-A+ | Freq. of Parent	P1/Q3: B 530_30-A+ , YG 610_20-A- | Freq. of Parent	P1/Q4: B 530_30-A- , YG 610_20-A- | Freq. of Parent
sg1_001.fcs	33.90%	0 %	0 %	0.17%	99.8 %
sg1_002.fcs	34.60%	0 %	0 %	0.12%	99.9 %
sg1_003.fcs	29.80%	0 %	0 %	0.17%	99.8 %
sg2_001.fcs	29.50%	0 %	0 %	0.05%	99.9 %
sg2_002.fcs	28.90%	$1.69x10^{-3}$ %	0 %	0.07%	99.9 %
sg2_003.fcs	30.0 %	0 %	0 %	0.07%	99.9 %
sg3_001.fcs	32.9 %	0 %	0 %	0.11%	99.9 %
sg3_002.fcs	34.0 %	0 %	0 %	0.11%	99.9 %
sg3_003.fcs	30.7 %	0 %	0 %	0.16%	99.8 %
sg12_001.fcs	31.3 %	0 %	0 %	0.10%	99.9 %
sg12_002.fcs	29.6 %	0 %	0 %	0.17%	99.8 %
sg12_003.fcs	29.4 %	0 %	0 %	0.12%	99.9 %
sg13_001.fcs	31.7 %	$1.73x10^{-3}$ %	0 %	0.14%	99.9 %
sg13_002.fcs	32.5 %	0 %	0 %	0.11%	99.9 %
sg13_003.fcs	27.4 %	$3.47x10^{-3}$ %	0 %	0.10%	99.9 %
sfEJ2-3.fcs	37.9 %	0 %	0 %	$5.47x10^{-3}$ %	100.0 %
	aEJ	SD			
vector	$5.47x10^{-3}$ %				
sg1	0.15%	0.000289			
sg2	0.06%	0.000096			
sg3	0.13%	0.000289			
Sg1+2 (30 bp)	0.13%	0.000369			
Sg1+3 (60 bp)	0.12%	0.000208			
